# Statistical Models for Infectious Diseases: A Useful Tool for Practical Decision-Making

**DOI:** 10.4269/ajtmh.19-0354

**Published:** 2019-05-28

**Authors:** Grant Brown, Marie Ozanne

**Affiliations:** Department of Biostatistics, University of Iowa, Iowa City, Iowa

The study of infectious diseases and the design of public health programs to combat them can be a source of seemingly endless complexity. When considering a disease such as visceral leishmaniasis, for example, questions remain about issues as fundamental as the human immune response, heterogeneity between pathogen and vector strains, the roles of vector and vertical transmission, the contribution of reservoir species, and interactions between all of these factors. Moreover, governmental and nongovernmental interventions must be implemented in the knowledge of this complexity, while also addressing a variable landscape of human preparedness and vulnerability. It is this latter task which is addressed in “Assessment of area-level disease control and surveillance vulnerabilities: an application to visceral leishmaniasis in Brazil,” in which the authors propose both a specific statistical approach to vulnerability estimation and a general argument for statistical inference in decision-making.^1^ They demonstrate the important ability of statistical models—simplified mathematical descriptions of the patterns or processes that give rise to the data we observe in real-world studies—to combine our knowledge about a subject with data to better understand important health problems.

The most familiar application of statistical modeling to infectious diseases, beyond basic procedures such as *t*-tests, concerns the direct modeling of infection processes. Most commonly, we define an acronym (e.g., SEIR) which is composed of discrete states that can be ascribed to the infection process (e.g., susceptible, exposed, infectious, and removed). These compartmental models describe the progression of individuals through these infection states and can be used to predict future infections, describe important pathogen characteristics, test the effectiveness of control measures, and design interventions. A foundational example of intervention evaluation and reproductive number estimation for statistical compartmental models, with a focus on the 1995 Ebola outbreak in the Democratic Republic of Congo, is given by Lekone and Finkenstädt.^2^ These tools help us to learn more from infectious disease data, and can be built to consider spatial data arising from surveillance efforts^3^ and nuanced individual-level information,^4^ and even to deal with the complexity of multiple pathogens or host/vector species. Although many in the field are at least passingly familiar with such techniques and their applications in forecasting, reproductive number estimation, and disease mapping, the “statistics” part is sometimes lost or even confused with deterministic, mathematical models.

Although modeling of infectious diseases has long been approached through mathematical models, such techniques are typically deterministic, ignoring randomness and variability. They generally provide a single estimate of the number of new infections at each time point, for instance, and do not account for any uncertainty in that estimate. By contrast, statistical infectious disease modeling techniques can provide evidence in terms of probability that an infection is spreading or being controlled in a population, that particular public health interventions are or are not having the desired impact, or whether infection rates vary by important demographic factors. These techniques are very general and can be modified to address innumerable hypotheses, provided appropriate data are available. For example, Upfill-Brown et al.^5^ provide a predictive risk model for poliovirus in Nigeria and describe steps for intervention resource targeting. Thus, although statistical techniques sometimes have additional implementation challenges, they confer clear advantages over mathematical models to inform policy. It is only through statistical techniques that we can formally weigh the evidence for particular phenomena.

In contrast to the modeling techniques described previously, Del Rio Vilas et al.^1^ address a different aspect of infectious disease modeling which is nevertheless critical and which has direct policy implications. They address the important topic of area vulnerability assessment: a task which is designed to aid in decision-making by inferring local capacity for disease control and surveillance efforts. Del Rio Vilas et al.^1^ identify several important challenges to vulnerability assessment and propose that their analysis addresses them. First, there exists uncertainty in such measures due both to missing data and to measurement error. Second, deterministic approaches, whether simple (e.g., a formula) or complex (e.g., resulting from an optimization algorithm) can be unstable and highly sensitive to outliers. Third, there is a need to account for varying population size. Fourth, there is a need for statistical inference; one must be able to communicate to stakeholders with confidence and with some explanation of uncertainty.

The approach of these authors, Bayesian spatial factor analysis, is an interesting method to address these concerns. In the same way that Bayesian compartmental models allow us to combine surveillance, laboratory, and expert opinions when considering the spread of infectious diseases, the Bayesian spatial factor analysis approach allows us to combine diverse (and often incomplete) data about public health capacity as they relate to disease over regions. At its core, this approach is a dimension reduction technique, summarizing many disparate measures in a simplified way, and it imposes a “smoothness” between neighboring regions. As such, it allows for the creation of summaries (see Figure 1 of Del Rio Vilas et al.^1^ in this issue) of multidimensional, noisy, and often missing capacity data. These results remain fairly straightforward to interpret and summarize, although still retaining a formal statistical/evidential interpretation. The authors also provide an illustration of the uncertainty associated with these estimates and note that it is quite large—an important consideration for any action taken on the basis of these results.

The approach discussed by Del Rio Vilas et al.^1^ can help target resources to regions at the highest risk, in the same way that compartmental models can evaluate intervention efforts and individual level models can help guide effective treatment of patients. Although the utility of these measures is clear, the authors do note some important limitations, particularly related to data quality and spatial scale. It is unsurprising that data are sometimes fragmentary or subject to large measurement error, but this limitation would hopefully be mitigated were these techniques to be more widely adopted by governmental organizations. The latter is also important to remember: health capacity can certainly vary on a small spatial scale, from neighborhood to neighborhood, and finer-grained data would be required to make data-driven decisions at that level. Nevertheless, the work demonstrates a natural approach to the development of vulnerability rankings and makes a good case for the increased use of statistical modeling in resource targeting and intervention development.
